# Lower leg muscle–tendon unit characteristics are related to marathon running performance

**DOI:** 10.1038/s41598-020-73742-5

**Published:** 2020-10-21

**Authors:** Bálint Kovács, István Kóbor, Zsolt Gyimes, Örs Sebestyén, József Tihanyi

**Affiliations:** 1 Department of Kinesiology, University of Physical Education, Alkotás u. 44, Budapest, 1123 Hungary; 2 Semmelweis University, MR Research Centre, Budapest, Hungary

**Keywords:** Musculoskeletal system, Muscle, Tendons

## Abstract

The human ankle joint and plantar flexor muscle–tendon unit play an important role in endurance running. It has been assumed that muscle and tendon interactions and their biomechanical behaviours depend on their morphological and architectural characteristics. We aimed to study how plantar flexor muscle characteristics influence marathon running performance and to determine whether there is any difference in the role of the soleus and gastrocnemii. The right lower leg of ten male distance runners was scanned with magnetic resonance imagining. The cross-sectional areas of the Achilles tendon, soleus, and lateral and medial gastrocnemius were measured, and the muscle volumes were calculated. Additional ultrasound scanning was used to estimate the fascicle length of each muscle to calculate the physiological cross-sectional area. Correlations were found between marathon running performance and soleus volume (r = 0.55, *p* = 0.048), soleus cross-sectional area (r = 0.57, *p* = 0.04), soleus physiological cross-sectional area (PCSA-IAAF r = 0.77, p < 0.01, CI± 0.28 to 0.94), Achilles tendon thickness (r = 0.65, *p* < 0.01), and soleus muscle-to-tendon ratio (r = 0.68, *p* = 0.03). None of the gastrocnemius characteristics were associated with marathon performance. We concluded that a larger soleus muscle with a thicker Achilles tendon is associated with better marathon performance. Based on these results, it can be concluded the morphological characteristics of the lower leg muscle–tendon unit correlate with running performance.

## Introduction

The human ankle plantar flexor muscles play a major role in producing propulsive force during endurance running^1–3^. The triceps surae muscle–tendon complex is equipped with a long compliant tendon and a strong and diverse muscle structure. It is a generally accepted concept that the Achilles tendon (AT) acts as a spring during running to store and return elastic energy and reduce the metabolic energy cost of the contractile element^4,5^. Running consists of a series of submaximal voluntary muscle contractions; thus, repetitive moderate force production is needed to propel the body forward. The magnitude of the contraction force depends on the running velocity and the motion of the lower leg. The energetic cost of contraction can be minimized if the fascicles are operating near the optimal length. During the early stance phase of running, the fascicles of the triceps surae operate under quasi-isometric conditions^6–9^; thus, a shorter fascicle with a low contraction velocity can result in a favourable contractile condition because shorter fascicles can maintain tension with low activation energy costs^10,11^. Therefore, the length change of the muscle–tendon complex mainly occurs in the tendon during the early stance phase of running^6,12,13^. Most elite marathon runners use rearfoot strike pattern^14^ where ankle joint flexion at stance is relevantly smaller^15^ resulting in less muscle–tendon unit lengthening compared to forefoot strike pattern. Because thicker tendon has a greater cross-sectional area (CSA), the acting force applying in greater surface, thus greater amount of force needed to stretch the tendon which increase the amount of stored elastic strain energy. Therefore it can store more elastic strain energy than a thin tendon at similar tendon extension because it has greater stiffness^16^. According to the literature, distance runners have thicker ATs than sprinters^17^ and non-runners^17–19^. However, it should be mentioned that mechanical properties of the tendons do not depend only on the morphological characteristics of the tendon. But the material and structure of the tendon also related to the mechanical properties of the tendon, too^20,21^. Thus, load induced changes in tendon material also can result in changes in the mechanical properties of the tendon^20,21^. To stretch a thicker tendon greater muscle force is required during the first half of the stance phase during running. We can assume that this force mainly produced by the soleus (SOL) because physiological cross-sectional area (PCSA) of the SOL is significantly larger than that of the gastrocnemii (GAS)^22,23^, and as a consequence, SOL produces three to four times greater positive work than GAS during running^12^. If we assume that SOL is generating at least twofold greater force than GAS during running then SOL contributes to elastic energy storage in the AT much more than the GAS muscles^6,7,24^, as well as to mechanical work^6,7,25^. Additionally, the SOL contains mainly slow twitch muscle fibres, and slow fibres lower the muscle volume-specific rate of energy use because slow muscles have lower rates of time-dependent cross-bridges^11^. Also, because GAS contains dominantly fast twitch fibers^26^ fatigue affects these muscles more, i.e. decreasing the mechanical output over time during running compared to SOL muscle^27–29^. Because muscle force generation capacity is related to CSA and the PCSA of the muscle^10,30,31^, greater force production could lead to morphological adaptations in the SOL. The mechanical properties of the tendons and muscles are influenced by their CSA and PCSA^10^; thus, the CSA and PCSA may have an impact on muscle–tendon interaction and consequently on running performance. However, this connection is not clear. Calculations with animal and cadaver muscles showed that there is an optimum PCSA/tendon cross-sectional area (tCSA) ratio^10,32^. Such a calculation has not been carried out on human triceps surae muscles in vivo thus far. Since the AT is the largest tendon in the human body, we may assume that the PCSA/tCSA ratio is different from the theoretical optimum and is greater for the SOL than for the GAS. If a thicker tendon is coupled with a shorter fascicle length and greater muscle stress, then the tendon stress also increases, and more elastic energy can be stored in the Achilles tendon due to the SOL force generation. To our knowledge, no previous report has investigated the correlation between triceps sure muscle morphology (i.e., CSA and PCSA) or the PCSA/tCSA ratio and running performance. Therefore, the purpose of this study was to test whether there is a link between morphological variables of triceps surae muscle tendon unit and marathon performance. Taking this information together, we hypothesized that runners who have a greater SOL PCSA, shorter fascicle length, thicker tendon and greater PCSA/tCSA ratio can complete the marathon distance in a shorter time (greater IAAF score). Additionally, we hypothesized that SOL morphological properties have a greater impact on running performance than GAS morphological properties.

## Methods

### Participants

Ten male marathon runners (mean and SD 29 ± 3.8 years, 177.1 ± 8.9 cm, 65.4 ± 5.8 kg) with a personal best International Amateur Athletic Federation (IAAF) score of 888.0 ± 184.0 (2 h 26 min on average) volunteered for this study. IAAF score points are used to classify running race time (performance) with a numerical value, which can be used for statistical analysis^33^. The runners had competed on international and national levels and had an average training volume of 120–200 km per week. All participants performed their best marathon race time within 2 years before this experiment. The scans were taken during the midseason. The participants had no musculoskeletal injury or pain in the lower extremities. All participants gave written informed consent to take part in the study, which was performed in accordance with the Declaration of Helsinki and was approved by the ethics committee of University of Physical Education (TE-KEB/No07/2018).

### Data collection

#### Magnetic resonance imagining scan

MRI images were taken from the right leg to measure morphological parameters of the triceps sure muscle tendon complex. A 3T Philips scanner (Ingenia 3.0T MRI system, Amsterdam, Netherlands) was used to acquire the MRI images. The runners were positioned supine, with neutral knee (180° between shank and thigh) and ankle joint angles (90° between foot and shank). A foam pad was placed below the calcaneus that elevated the leg slightly and prevented weight-induced deformation of the muscle during the scan. The scans were performed using a T1-weighted turbo spin echo sequence (slice thickness = 5 mm, slice gap = 0 mm, slice scan order: interleaved, TR = 650 ms, TE = 20) for all measurements. Because of the limited field of view of the probe (FOV = 40 cm), the images were taken in two parts to ensure that the records contained the origin and insertion of the plantar flexor muscle–tendon complex. The overlapping images were manually removed from the analysis. The axes during the MRI image acquisition was set carefully to align as possible as it can with the muscle–tendon unit.

#### Architectural measurement

An additional ultrasound measurement was applied to estimate the muscle architecture of the SOL, medial gastrocnemius (MG) and lateral gastrocnemius (LG) (6 cm field of view, B-mode linear array probe, 13 MHz scanning frequency, Hitachi-Aloka EUB 405 plus, Japan). Participants were laid prone on a table with a neutral ankle and knee joint position. Acoustic gel was applied between the skin and the probe, which was placed at approximately 50% of the length of each muscle, but the locations were optimized for fascicle imaging^34^. The probe was placed manually on the skin and held carefully over the skin to avoid applying too much pressure to the tissues underneath.

### Data analysis

#### Magnetic resonance image processing

**Figure 1:**
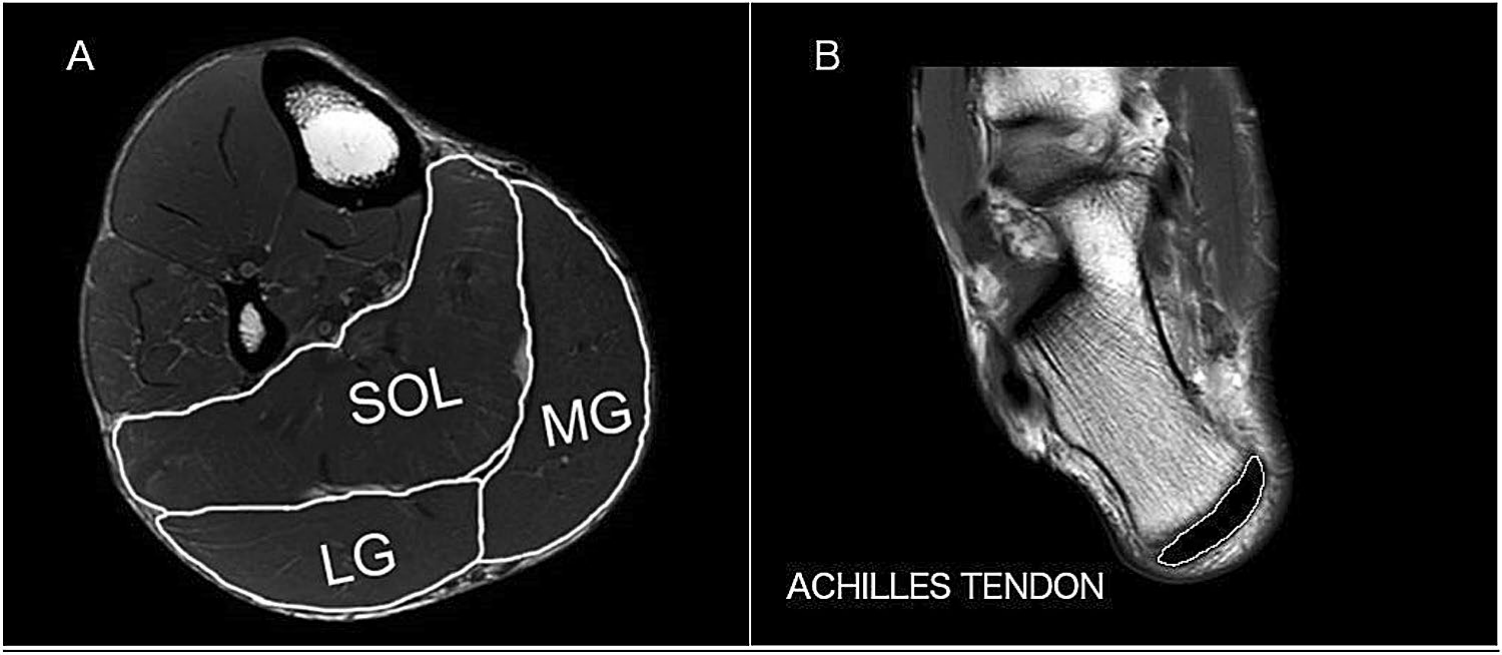
Representative magnetic resonance image from the middle of the lower leg for the calculation cross-sectional areas (**A**). The triceps surae compartments were separately outlined manually. (**B**) A sample image of the maximal distal Achilles tendon. Each segmented area (*SOL* soleus, *MG* medial gastrocnemius, *LG* lateral gastrocnemius) marked with white line.

The images were analysed using ImageJ 1.44b (National Institutes of Health, USA). The CSA of each muscle and tendon was manually outlined on all of scans that the muscles and tendon were visible on and then the area was measured (Fig. 1). The images were analysed by two separate raters. All segmentations were checked by a researcher (author IK) experienced in studying and measuring MRI scans. The test–retest procedure was applied to estimate the reliability of the CSA measurements. Each CSA measured by the two raters was averaged and then used to calculate muscle volume and mass. The lengths of the muscles and AT were calculated by summing the number of analysed slices and multiplied by 0.5. The total volume of the plantar flexor muscles and AT was calculated by summing the volume of each slice, i.e., the product of slice area and slice thickness (0.5 cm)^23,30,35^. Muscle mass was calculated as muscle volume multiplied by muscle density (1.056 g/cm^3^)^36^. The PCSA was calculated by dividing muscle volume by fascicle length.

#### Ultrasound image processing

**Figure 2:**
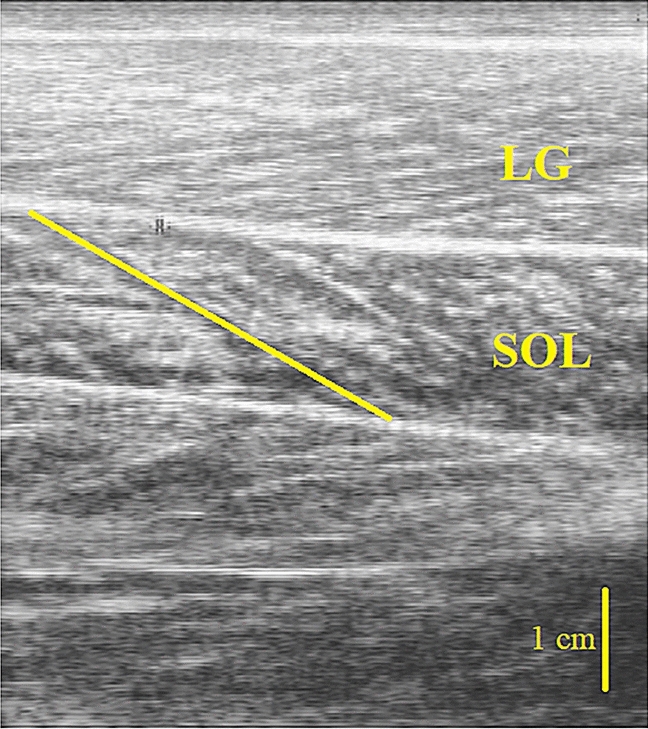
Representative ultrasound image of soleus in sagittal plane for estimate fascicle length. The image was taken at 50% of the muscle length because that region possibly contains the longest fascicles of the muscles. Fascicle length (solid yellow line along fascicles), are drawn in the images.

The longest fascicle was outlined manually in each image, and then the length of the line was measured. If needed, multiple lines were drawn to follow the curvature of the fascicle^37^ (Fig. 2). If part of the fascicles was outside the field of view, fascicle length was estimated by linear extrapolation. The image analysis for muscle architecture was performed in ImageJ 1.44b (National Institutes of Health, USA).

#### Statistics

Data are presented as the means and standard deviations. Because of the small sample size, the Shapiro–Wilk normality test was used to test the normality of the data. To determine the relative between-rater reliability of each muscle and tendon, an intraclass correlation coefficient (ICC) was calculated using a two-way mixed-effects model (average measures), along with the upper and lower 95% confidence interval (CI±). The ICC estimate was considered good between 0.75 and 0.9 and excellent above 0.9^38^. A Bland–Altman plot was used to determine the bias between the raters and the limits of agreement (see Supplementary material). Pearson correlations were calculated to investigate the relationship between marathon performance and the properties of muscles and tendons. The magnitude of significant correlations was quantified using the thresholds recommended by Hopkins ^39^, i.e., 0–0.1 as small, 0.1–0.3 as moderate, 0.3–0.5 as large, 0.5–0.7 as very large and 0.9–1 as extremely large correlations. Additionally, the 95% confidence intervals for each corresponding Pearson coefficient were calculated. In cases of non-Gaussian data distributions, a Spearman rank correlation was used. All statistical calculations were performed using SPSS (SPSS Inc., Chicago, IL, USA v. 25), and statistical significance was set at an alpha level of 0.05.

## Results

**Figure 3:**
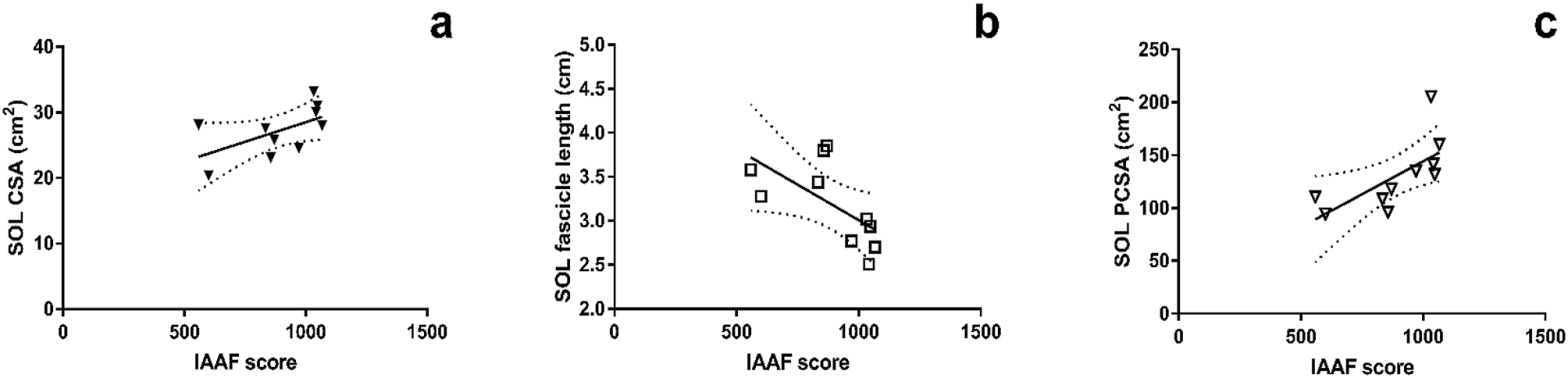
Correlation between IAAF and (**a**) soleus maximal cross-sectional are (r = 0.57, CI − 0.08 to 0.88, *p* = 0.041), (**b**) soleus fascicle length (r = − 0.63, p = 0.02, CI± − 0.90 to − 0.001), and (**c**) soleus physiological cross-sectional area (PCSA-IAAF r = 0.77, p < 0.01, CI± 0.28 to 0.94).

**Figure 4:**
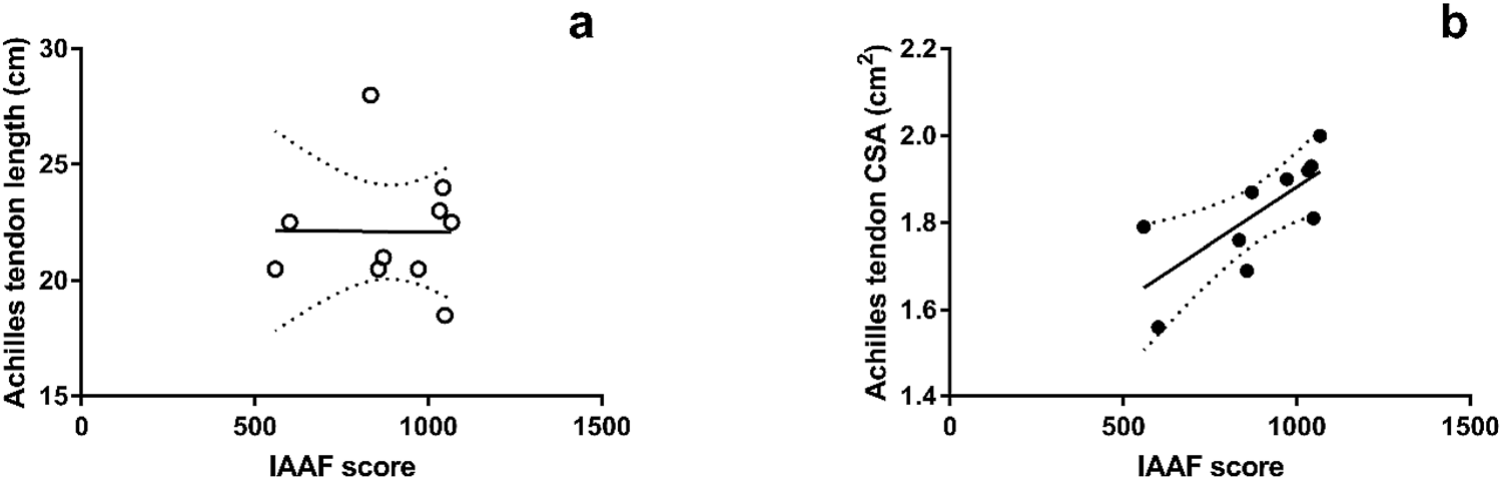
Correlation between IAAF and (**a**) Achilles tendon length (r = − 0.01, *p* = 0.48 CI± − 0.63 to 0.62) (**b)** Achilles tendon maximal cross-sectional area (r = 0.65, p = 0.01, CI± 0.04 to 0.91).

**Figure 5:**
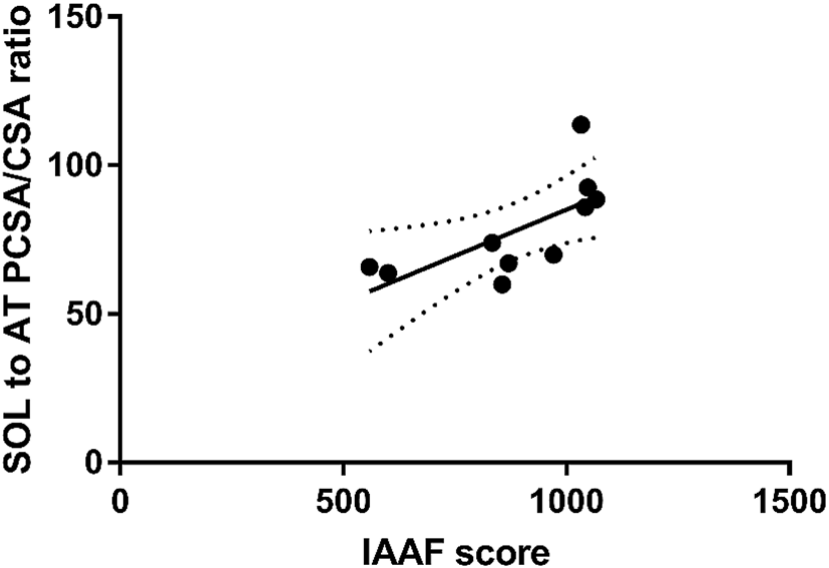
Correlation between IAAF score (representing marathon performance) and ratio of soleus physiological cross-sectional area to Achilles tendon cross-sectional area. There is a large correlation (r = 0.68, *p* = 0.029 CI± 0.09 to 0.92) between these variables.

No architectural (fascicle length) or morphological (volume, CSA, PCSA) parameters of LG and MG correlated with marathon performance. On the other hand, marathon performance correlated with maximal CSA (r = 0.57; *p* = 0.041 CI± 0.08 to 0.88) and volume of the SOL (r = 0.55; *p* = 0.048, CI± 0.11 to 0.87). SOL muscle fascicle length negatively correlated with marathon performance (r = − 0.63, *p* = 0.02, CI± − 0.90 to − 0.001) (Fig. 3). PCSA of the SOL showed large positive correlation with marathon performance (r = 0.77, *p* < 0.01, CI± 0.28 to 0.94) (Fig. 3). The total PCSA of triceps surae also showed large positive correlation with marathon performance (r = 0.72, *p* < 0.009, CI± 0.16 to 0.93). The maximal CSA of AT (r = 0.65, p = 0.01, CI± 0.04 to 0.91) correlated with marathon performance (Fig. 4). The largest distal CSA of the AT also correlated with marathon performance (r = 0.65, *p* = 0.02). There is a positive correlation between SOL PCSA and tCSA (r = 0.61, *p* = 0.029) suggesting that those who have large SOL more likely to have thick AT in absolute term. The SOL PCSA/tCSA ratio also correlated with marathon performance (r = 0.68, *p* = 0.029, CI± 0.09–0.92) (Fig. 5). The mean and SD values of the plantar flexor muscle tendon unit properties are listed in Table 1. The calculated SOL volume was 48.89%, that of the MG was 31.66%, and that of the LG was 19.44% of the total triceps surae volume. The PCSA of the SOL was threefold greater than that of the MG and fourfold greater than that of the LG, and the SOL possessed 60.12% of the total PCSA of the triceps surae.

**Table 1:** The measured and calculated (mean and SD) morphological parameters of the triceps surae muscle–tendon complex.

	Achilles tendon	Soleus	Medial gastrocnemius	Lateral gastrocnemius
Length (cm)	22.10 ± 2.61	32.60 ± 2.89	25.95 ± 2.66	24.20 ± 2.14
Fascicle length (cm)	–	3.18 ± 0.47	5.24 ± 0.72	5.37 ± 0.84
Volume (cm^3^)	0.53 ± 0.07	452.9 ± 88.24	293.3 ± 69.78	180.1 ± 25.81
Muscle mass (g)	–	487.3 ± 93.18	309.8 ± 73.68	190.1 ± 27.25
CSA (cm^2^)	1.82 ± 0.13	27.16 ± 3.82	18.96 ± 3.34	13.31 ± 2.11
AT distal CSA (cm^2^)	1.20 ± 0.11	–	–	–
PCSA (cm^2^)	–	130.3 ± 33.59	60.04 ± 15.72	35.88 ± 5.73
muscle to AT volume ratio	–	858.44 ± 150.47	550.70 ± 92.63	343.68 ± 59.88
Muscle PCSA to AT ratio	–	78.12 ± 16.79	30.79 ± 8.0	18.39 ± 2.68

*CSA* cross sectional area, *AT* Achilles tendon, *PCSA* physiological cross-sectional area.

The results of the correlation analysis are summarized in Table 2. The results of the interrater reliability test showed excellent ICC values for all muscles and the tendon (see supplementary material).

**Table 2:** Correlation coefficients between marathon running performance and triceps surae muscle–tendon morphological characteristics.

Variables	Achilles tendon			Soleus			Lateral gastrocnemius			Medial gastrocnemius		
	r	*p*	95% CI±	r	*p*	95% CI±	r	*p*	95% CI±	r	*p*	95% CI±
Length	− 0.01	0.48	− 0.63 to 0.62	0.34	0.16	− 0.36 to 0.79	0.12	0.36	− 0.54 to 0.69	0.21	0.27	− 0.47 to 0.74
Volume	0.32	0.18	− 0.38 to 0.79	0.55	0.048	0.11 to 0.87	0.06	0.43	− 0.59 to 0.66	0.41	0.11	− 0.28 to 0.82
CSA max	**0.65**	**0.01**	**0.04 to 0.91**	**0.57**	**0.04**	**0.08 to 0.88**	0.05	0.44	− 0.59 to 0.66	0.37	0.14	− 0.33 to 0.81
PCSA	–	–	–	**0.77**	**0.01**	**0.28 to 0.94**	0.36	0.29	− 0.33 to 0.81	0.32	0.35	− 0.37 to 0.79
Fascicle length	–	–	–	**− 0.63**	**0.02**	**− 0.90 to − 0.01**	− 0.26	0.22	− 0.76 to 0.43	− 0.02	0.47	− 0.64 to 0.61

The corresponding *p* value and 95% confident interval was calculated as well. Bold numbers indicate significant correlations.

## Discussion

The purpose of this study was to investigate if there is correlation between the morphological and architectural characteristics of triceps surae muscle–tendon unit and running performance. We hypothesized that faster marathon runners have greater PCSAs and shorter fascicle lengths in the SOL and thicker ATs than slower marathon runners. We found a positive correlation between IAAF score and the PCSA of the SOL and a negative correlation between IAAF score and the fascicle length of the SOL. Additionally, a thicker AT was linked to a better IAAF score; therefore, our results showed that the morphology of the SOL PCSA and tCSA correlate with marathon performance. This novel finding might supports the concept that the SOL plays a more important role in endurance running than the GAS muscles^6,7,40^.

The MRI-based morphological parameters of the muscle structures (CSA, volume) are in alignment with those from previous reports^23,41–43^. The fascicle lengths estimated from the ultrasound images are similar to the findings of earlier studies^9,37,40,42–45^; thus, the calculated PCSA is also similar to that from previous reports^23,42,43^. As expected, we found that the SOL had a greater muscle volume, CSA, and PCSA and a greater PCSA/tCSA ratio than the GAS muscles. This can explain why the SOL muscle produces greater force and positive work than the GAS during moderate-pace running^6,7,11,12^ assuming that SOL and GAS are to shortening the same amounts. However, a greater force production often pairs with greater metabolic demand of the contractile elements in general, but muscle fibre composition (i.e., predominance of slow twitch fibres) can compensate for this effect^26^. It is known that the SOL primarily contains slow twitch muscle fibres^26^ and that these fibres have a lower muscle volume-specific rate of energy demand since slow muscles have lower rates of time-dependent cross-bridges^46^.

We found that runners with greater IAAF scores had shorter SOL fascicles, possibly because muscles with shorter fascicles work more economically because they involve a smaller active volume of muscle, and therefore, a smaller amount of ATP is consumed^11^. The decreased muscle metabolic energy demand can lead to a decreased cost of running as well; thus, it can improve running performance and possibly running economy.

The function of the AT can also decrease the metabolic energy cost of the contractile elements. It has been shown that the function of the tendon depends on the morphological characteristics of the tendon^17,19,47^. Since the CSA of the AT is different at each AT length, it seems important to select the appropriate CSAs that may influence running performance. Magnusson and Krajer^47^ reported that CSAs measured one centimetre above the calcaneal insertion showed the largest difference between runners and non-runners. In contrast, Ueno et al.^17^ found that the AT CSA of distance runners was significantly larger than that of sprinters and non-runners only when the CSA below the SOL-tendon junction was selected for comparison. However, in a later study, Ueno et al.^19^ did not find a significant correlation between distal AT CSA and running economy or running performance. In our study, we correlated both the distal and proximal AT CSA with marathon performance and found a significant association between the two variables, indicating that a thicker AT is beneficial for running the marathon distance in a shorter time. It is difficult to resolve the contradiction between our results and those of Ueno et al.^19^. It can be assumed that AT length has a greater impact on 5000-m running performance than AT CSA since Ueno et al.^19^ reported a significant relationship between MG tendon length and running economy. From this point of view, we can imagine that there may be a difference in ankle kinetics and kinematics when running long or short distances. This assumption is supported by our results; namely, we did not find an association between AT length and marathon running performance. The average running speed of our runners during marathon is 5.01 ms^−1^ which is obviously less compared to elite 5000 m runners racing speed (14 min race time equal to 6.11 ms^−1^ running speed). Because foot strike pattern seems to be influenced by running velocity (especially above 5 ms^−1^)^15,48^ and footwear^49^ i.e. track runners usually wearing (light weighted and thin) spike shoes thus, we can assume that shorter track runners possibly use forefoot strike pattern. But on the other hand, the majority of elite marathon runners are using rearfoot strike pattern^14^. Kinematic difference between rearfoot and forefoot strike pattern has been demonstrated^15,27,28^ showing that ankle joint flexion is greater during forefoot strike which lead to a greater muscle tendon unit lengthening as well. In that case a thin tendon would be better since greater elastic energy could be stored by applying smaller muscle force compared to a thick tendon.

To the best of our knowledge, nobody has studied how the triceps surae muscles and tCSA ratio can be related to running performance, especially marathon running time. Even though experiments and calculations suggest that there is an optimum muscle-to-tendon area ratio that may minimize muscle–tendon mass and help deliver greater mechanical energy via the muscle–tendon system^32,50^. Theoretically, the optimum ratio is 34^10^, which reduces the incidence of tendon damage and enables the muscle–tendon complex to perform more mechanical work. We found that only the MG PCSA/tCSA ratio approached this value; the LG PCSA/tCSA ratio was considerably less, and the soleus PCSA/tCSA ratio was more than twice the theoretical optimum. Ker et al.^10^ argued that a thinner tendon requires longer fascicles to be able to shorten more. We found that fascicles in the SOL muscle are short, which contradicts this theory. However, this construction can be beneficial, especially during stretch–shortening muscle contraction. Short fascicles relative to muscle length result in large PCSAs and, as a consequence, greater force generation capacity. Because the SOL PCSA was found to be largest in our study, the SOL presumably had the capacity to exert greater force; therefore, the SOL AT was subjected to a larger stress, which assumes a larger elastic energy storage capacity in the tendon during the ankle joint flexion phase of running. It has been reported that the SOL AT length is three times shorter than that of GL and GM^19^, but the distal tCSA presumably is the same. Since tendon stiffness depends on both the tendon length and CSA primarily, it is possible that SOL AT stiffness could potentially be greater than GM and GL tendon stiffness; in other words, the SOL has a greater contribution to tendon stiffness than the GAS. Because stiffness is related to running performance^51,52^, we may conclude that the SOL PCSA/tCSA ratio has a prominent role in better performance in marathon running. The correlation between SOL PCSA/tCSA ratio and IAAF score may indicate that a large SOL PCSA with thin AT correlate with better marathon race time. However, this correlation must be considered in relative term. It is unlikely that those who have large SOL muscle also would have thin tendon. We found a strong positive correlation between SOL PCSA and AT CSA suggesting that those who have large SOL more likely to have a thick AT in absolute term.

This study has some limitations that must be addressed. The participants performed their personal best in the previous 2 years; thus, the current performance level was not taken into consideration. However, the athletes were regularly training during this period and reported no weight changes over this period, so we can assume that no remarkable changes occurred in their lower leg morphology. It must be noted that the limited size of the sample rases some concern about generalizing the conclusion. Extreme outlier data points could have strongly affected the magnitude and direction of our correlation analysis. The statistical method we used, prove no causality only correlation between the selected variables which must be considered when interpreting the results of this study. In the present study, we did not examine the mechanical properties of the AT and plantar flexor muscles; therefore, the relationships between the mechanical characteristics and morphological properties of the plantar flexor muscle–tendon unit and remain unclear.

In summary, we found that the soleus PCSA/tCSA ratio is much greater than the theoretical optimum and that a greater ratio resulted in a shorter marathon running time. From a better running performance point of view, a large PCSA of the soleus muscle and a thick Achilles tendon are beneficial because more elastic energy can presumably be stored (and recoiled) in the AT, which enables runners to run more efficiently. From our results, we can draw conclusions in accordance with our hypothesis that morphological and architectural characteristics (of the triceps surae and AT) correlate with running performance. In addition, our results allow us to conclude that in marathons running, the soleus has a more significant role than the gastrocnemius muscle.

## Supplementary information

Supplementary Information 1.

Supplementary Information 2.
