# Supplementary material for: Lower leg muscle–tendon unit characteristics are related to marathon running performance
Source: Sci Rep. 2020 Oct 21;10:17870. doi: 10.1038/s41598-020-73742-5 (PMC7578824; doi:10.1038/s41598-020-73742-5)
Supplement: Supplementary file 2 — Supplementary Information 2. [file 41598_2020_73742_MOESM2_ESM.docx]

**Lower leg muscle-tendon unit characteristics are related to marathon running performance**

Authors: Bálint Kovács, István Kóbor, Zsolt Gyimes, Örs Sebestyén, József Tihanyi

Reliability tests:

Achilles Tendon

**A**

| **Case Processing Summary** | | | |
| --- | --- | --- | --- |
|  | | N | % |
| Cases | Valid | 442 | 67,8 |
|  | Excluded^a^ | 210 | 32,2 |
|  | Total | 652 | 100,0 |
| a. Listwise deletion based on all variables in the procedure. | | | |

| **Reliability Statistics** | |
| --- | --- |
| Cronbach's Alpha | N of Items |
| ,997 | 2 |

| **B**  **Intraclass Correlation Coefficient** | | | | | | | |
| --- | --- | --- | --- | --- | --- | --- | --- |
|  | Intraclass Correlation^b^ | 95% Confidence Interval | | F Test with True Value 0 | | | |
|  |  | Lower Bound | Upper Bound | Value | df1 | df2 | Sig |
| Single Measures | ,995^a^ | ,993 | ,995 | 362,647 | 441 | 441 | ,000 |
| Average Measures | ,997^c^ | ,997 | ,998 | 362,647 | 441 | 441 | ,000 |


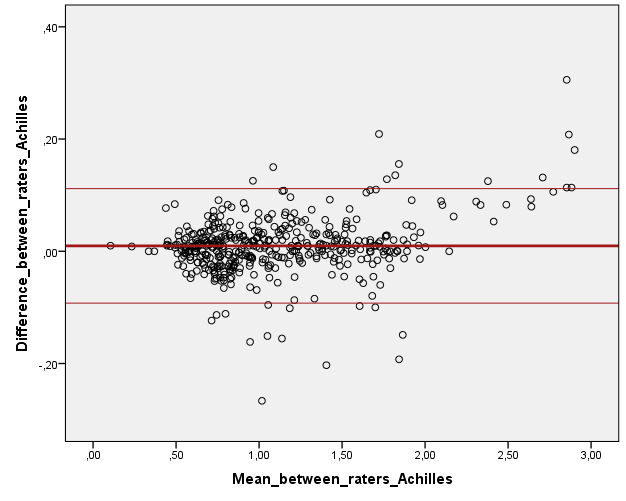
**C**

Figure 1. Comparisons between the two raters measurements on Achilles tendon. Table A shows the descriptive results of the reliability test, and table B shows results of the reliability test. Results are illustrated (C) by differences between pairs of measurements as a function of the mean measurements. Solid and thin red lines depict bias and 95% limits of agreement, respectively.

SOL

**A**

| **Case Processing Summary** | | | |
| --- | --- | --- | --- |
|  | | N | % |
| Cases | Valid | 652 | 100,0 |
|  | Excluded^a^ | 0 | ,0 |
|  | Total | 652 | 100,0 |
| a. Listwise deletion based on all variables in the procedure. | | | |

| **Reliability Statistics** | |
| --- | --- |
| Cronbach's Alpha | N of Items |
| ,999 | 2 |

| **B**  **Intraclass Correlation Coefficient** | | | | | | | |
| --- | --- | --- | --- | --- | --- | --- | --- |
|  | Intraclass Correlation^b^ | 95% Confidence Interval | | F Test with True Value 0 | | | |
|  |  | Lower Bound | Upper Bound | Value | df1 | df2 | Sig |
| Single Measures | ,998^a^ | ,998 | ,999 | 1197,892 | 651 | 651 | ,000 |
| Average Measures | ,999^c^ | ,999 | ,999 | 1197,892 | 651 | 651 | ,000 |
| 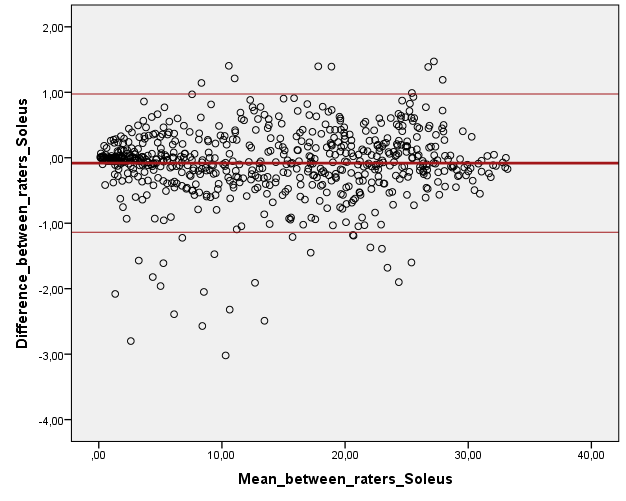**C**  Figure 2 Comparisons between the two raters measurements on soleus muscle. Table A shows the descriptive results of the reliability test, and table B shows results of the reliability test. Results are illustrated (C) by differences between pairs of measurements as a function of the mean measurements. Solid and thin red lines depict bias and 95% limits of agreement, respectively. | | | | | | | |
| MG  **A**   \| **Case Processing Summary** \| \| \| \| \| --- \| --- \| --- \| --- \| \|  \| \| N \| % \| \| Cases \| Valid \| 519 \| 79,6 \| \| Excluded^a^ \| 133 \| 20,4 \| \| Total \| 652 \| 100,0 \| \| a. Listwise deletion based on all variables in the procedure. \| \| \| \|  \| **Reliability Statistics** \| \| \| --- \| --- \| \| Cronbach's Alpha \| N of Items \| \| ,999 \| 2 \|  \| **B**  **Intraclass Correlation Coefficient** \| \| \| \| \| \| \| \| \| --- \| --- \| --- \| --- \| --- \| --- \| --- \| --- \| \|  \| Intraclass Correlation^b^ \| 95% Confidence Interval \| \| F Test with True Value 0 \| \| \| \| \| Lower Bound \| Upper Bound \| Value \| df1 \| df2 \| Sig \| \| Single Measures \| ,997^a^ \| ,997 \| ,998 \| 755,434 \| 518 \| 518 \| ,000 \| \| Average Measures \| ,999^c^ \| ,998 \| ,999 \| 755,434 \| 518 \| 518 \| ,000 \|   **C**  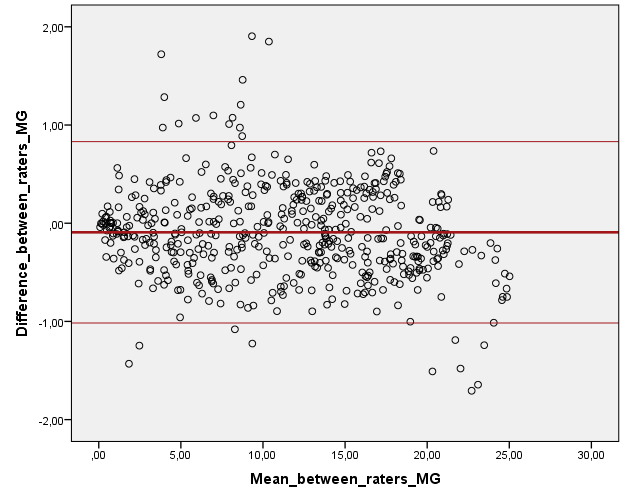 | | | | | | | |
| Figure 3 Comparisons between the two raters measurements on medial gastrocnemius muscle. Table A shows the descriptive results of the reliability test, and table B shows results of the reliability test. Results are illustrated (C) by differences between pairs of measurements as a function of the mean measurements. Solid and thin red lines depict bias and 95% limits of agreement, respectively. | | | | | | | |

LG

**A**

| **Case Processing Summary** | | | |
| --- | --- | --- | --- |
|  | | N | % |
| Cases | Valid | 484 | 74,2 |
|  | Excluded^a^ | 168 | 25,8 |
|  | Total | 652 | 100,0 |
| a. Listwise deletion based on all variables in the procedure. | | | |

| **Reliability Statistics** | |
| --- | --- |
| Cronbach's Alpha | N of Items |
| ,998 | 2 |

| **B**  **Intraclass Correlation Coefficient** | | | | | | | |
| --- | --- | --- | --- | --- | --- | --- | --- |
|  | Intraclass Correlation^b^ | 95% Confidence Interval | | F Test with True Value 0 | | | |
|  |  | Lower Bound | Upper Bound | Value | df1 | df2 | Sig |
| Single Measures | ,996^a^ | ,995 | ,996 | 455,770 | 483 | 483 | ,000 |
| Average Measures | ,998^c^ | ,997 | ,998 | 455,770 | 483 | 483 | ,000 |
| **C**  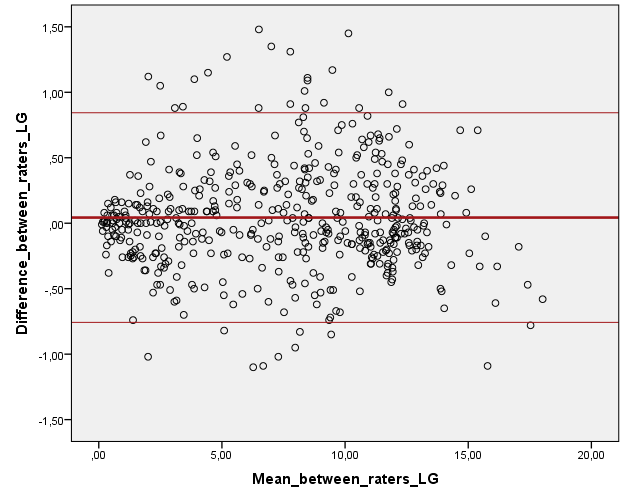Figure 4. Comparisons between the two raters measurements on lateral gastrocnemius muscle. Table A shows the descriptive results of the reliability test, and table B shows results of the reliability test. Results are illustrated (C) by differences between pairs of measurements as a function of the mean measurements. Solid and thin red lines depict bias and 95% limits of agreement, respectively. | | | | | | | |


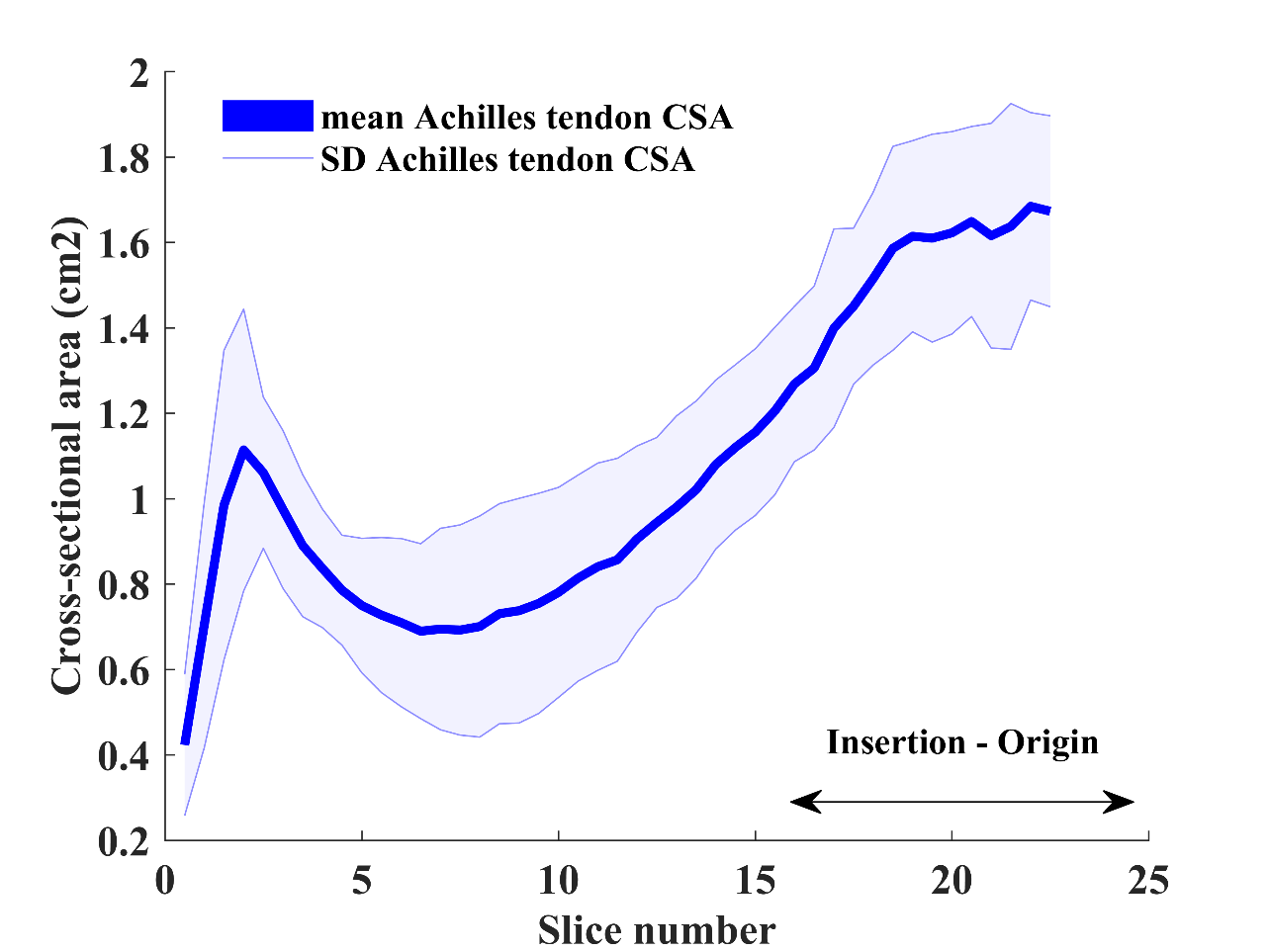


Figure 5. Cross-sectional area along the length of the Achilles tendon (mean and standard deviation). Scan 1 is the first appearance of the Achilles tendon above the calcaneal tuberosity. The last section is the junction point with medial gastrocnemius. Data are means and SD (n=10).
